# Supplementary material for: Comparative meta-analysis of cold snare polypectomy and endoscopic mucosal resection for colorectal polyps: assessing efficacy and safety
Source: PeerJ. 2024 Dec 19;12:e18757. doi: 10.7717/peerj.18757 (PMC11663405; doi:10.7717/peerj.18757)
Supplement: Supplemental Information 2 [file peerj-12-18757-s002.docx]

**Supplemental Article 1** Search strategy

**PubMed**

(((Colon) OR ((((((((((colorectal) ) OR (colonic)) OR (rectal)) OR (rectum)) OR (colorectum)) OR (intestinal)) OR (intestine)) OR (colo-rectal)) OR (sigmoid))) AND ((((Cold snare) OR (cold snaring)) OR (cold polypectomy)) OR (cold snare polypectomy))) AND ((Endoscopic Mucosal Resection) OR ((((((Endoscopic Mucosal Resections) OR (Mucosal Resection, Endoscopic)) OR (Resection, Endoscopic Mucosal)) OR (EMR)) OR (endoscopic aspiration mucosectomy)) OR (endoscopic mucosectomy)))

**Web of Science**

#1 ((((((((((TS=(colon)) OR TS=(colorectal))) OR TS=(colonic)) OR TS=(rectal )) OR TS=(rectum )) OR TS=(colorectum )) OR TS=(intestinal )) OR TS=(intestine )) OR TS=(colo-rectal)) OR TS=(sigmoid)

#2 ((((((TS=(endoscopic mucosal resection)) OR TS=(Endoscopic Mucosal Resections)) OR TS=(Mucosal Resection, Endoscopic)) OR TS=(Resection, Endoscopic Mucosal)) OR TS=(EMR)) OR TS=(endoscopic aspiration mucosectomy)) OR TS=(endoscopic mucosectomy)

#3 (((TS=(Cold snare)) OR TS=(cold snaring)) OR TS=(cold polypectomy)) OR TS=(cold snare polypectomy)

#4 #3 AND #2 AND #1

**EMBASE**

#1 'endoscopic mucosal resection'/exp OR 'endoscopic mucosal resection' OR (endoscopic AND mucosal AND resections) OR (mucosal AND resection, AND endoscopic) OR (resection, AND endoscopic AND mucosal) OR emr OR (endoscopic AND aspiration AND mucosectomy) OR (endoscopic AND mucosectomy)

#2 'colon'/exp OR colon OR colorectal OR colonic OR rectal OR rectum OR colorectum OR intestinal OR intestine OR 'colo rectal' OR sigmoid

#3 'cold snare polypectomy'/exp OR 'cold snare polypectomy' OR (cold AND snare) OR (cold AND snaring) OR 'cold polypectomy'

#4 #1 AND #2 AND #3

**CINAHL**

S1 TX colon OR TX colorectal OR TX colonic OR TX rectal OR TX rectum OR TX colorectum OR TX intestinal OR TX intestine OR TX colo-rectal OR TX sigmoid

S2 TX Cold snare OR TX cold snaring OR TX cold polypectomy OR cold snare polypectomy

S3 TX endoscopic mucosal resection OR TX Endoscopic Mucosal Resections OR TX Mucosal Resection, Endoscopic OR TX Resection, Endoscopic Mucosal OR TX EMR OR TX endoscopic aspiration mucosectomy OR endoscopic mucosectomy

S4 S1 AND S2 AND S3

**Cochrane Library**

ID Search

#1 MeSH descriptor: [Colon] explode all trees

#2 MeSH descriptor: [Rectum] explode all trees

#3 MeSH descriptor: [Intestines] explode all trees

#4 MeSH descriptor: [Colon, Sigmoid] explode all trees

#5 (colorectal):ti,ab,kw OR (colonic):ti,ab,kw OR (rectal):ti,ab,kw OR (colorectum):ti,ab,kw OR (intestinal):ti,ab,kw

#6 (intestine):ti,ab,kw OR (colo-rectal):ti,ab,kw

#7 #1 or #2 or #3 or #4 or #5 or #6

#8 MeSH descriptor: [Endoscopic Mucosal Resection] explode all trees

#9 (Endoscopic Mucosal Resections):ti,ab,kw OR (Mucosal Resection, Endoscopic):ti,ab,kw OR (Resection, Endoscopic Mucosal):ti,ab,kw OR (EMR):ti,ab,kw OR (endoscopic aspiration mucosectomy):ti,ab,kw

#10 endoscopic mucosectomy

#11 #8 or #9 or #10

#12 (Cold snare):ti,ab,kw OR (cold snaring):ti,ab,kw OR (cold polypectomy):ti,ab,kw OR (cold snare polypectomy):ti,ab,kw

#13 #7 and #11 and #12
