# Supplementary material for: Comparative meta-analysis of cold snare polypectomy and endoscopic mucosal resection for colorectal polyps: assessing efficacy and safety
Source: PeerJ. 2024 Dec 19;12:e18757. doi: 10.7717/peerj.18757 (PMC11663405; doi:10.7717/peerj.18757)
Supplement: Supplemental Information 3 [file peerj-12-18757-s003.docx]

The rationale for conducting the systematic review / meta-analysis:

The two most prevalent techniques for treating colorectal polyps are cold snare polypectomy (CSP) and endoscopic mucosal resection (EMR). Studies have compared their safety and efficacy, but there are variations in their research methodologies, including patient selection criteria, assessment methods, the type of healthcare professional performing the treatment, and more. Therefore, a systematic review and meta-analysis are essential to comprehensively analysing current literature results, synthesising information, guiding clinical decision-making, and potentially improving therapeutic outcomes. This study specifically focuses on comparing the complete resection rate, en bloc rate, perforation, delayed bleeding, and procedure time.

The contribution that it makes to knowledge in light of previously published related reports, including other meta-analyses and systematic reviews:

Existing studies have mainly compared endoscopic mucosal resection (EMR) to endoscopic submucosal dissection (ESD) or cold snare polypectomy (CSP) to hot snare polypectomy (HSP), lacking direct CSP vs. EMR comparisons. As an example, studies focused on the comparison of R0 and en bloc resection rates of EMR and CSP cannot be found. In contrast, our meta-analysis provides detailed comparisons of polyp removal techniques, including complete resection rate, en bloc rate, perforation, delayed bleeding, and procedure time for CSP vs. EMR, offering clinical insights.
